# Infantile Stools and Their Significance

**Published:** 1898-03

**Authors:** 


### INFANTILE STOOLS AND THEIR SIGNIFICANCE.

Charles Douglas, M. D., contributes a valuable paper with this title to the *Physician and Surgeon.* He says:

Green Stools, what are they? It is difficult to fully realize all the disturbing factors which result in this colored stool. As two healthy parties, mother and child, always contribute to and produce a healthy yellow stool, we must have an unhealthy influence in either one or other to produce a different result. Writers on this subject explain this green color by attributing it to the presence of chromogenic bacteria; but this is unsatisfactory, and does not explain the cause of disturbance. When we look at the great varieties of green stools, from the occasional one produced by the milk of a tired or irritated mother, in a very healthy child, to the greenish gray, foul, pasty one of the extremely rachitic child, we realize that numerous factors must intervene to produce all these shades in color, consistence and odor. Certain it is that the character of the bacteria is quickly changed, or their lives and number are rapidly reduced either by a diet suitable to the child, or healthy gastric juices in the child.

When we consider the different types of green stool in color, odor, and consistence—from the light green to the deep chrome hue; from the inodorous to the extremely foul and rotten; and from the thick, slimy, to the extremely liquid—we can not but believe that very different conditions must be present in the ailmentary canal to explain these results, aside from bacteria.

The deep chrome hue as against the lighter color in the same consistence of stool would indicate a greater food disturbance if the color is due to bile. In other words, there would be a less effort at digestion.

The extremely foul stool would show a marked albuminous decomposition, while the absence of odor would indicate healthy digestion, and where the stools were very liquid, extreme vermicular activity.

The very slimy stool would indicate a catarrhal condition, more or less severe and extensive, and generally mild in character, when compared with the profuse, watery, exosmotic discharges caused by an acute milk infection.

Whatever the character of the green stool may be, we see that it indicates an unhealthy condition in the digestive process, and it should receive our careful attention. The green color without other change shows the unsuitable dietary and nature's rapid efforts to remove it, quickly carrying the bile and imperfectly digested food out, thus checking nutrition and wasting secretions. The foul stools show the putrefactive process consuming the dietary, thus not only robbing the body of dietetic suppport but often loading the blood with these putrefactive germs through their absorption. The slimy and thin, watery stools by reflex action not only check digestion, but also consume the albumin and fibrin of the blood itself in direct proportion with their size and frequency.

From these facts we realize that green stools are errors or due to errors; that they always interfere with perfect alimentation; that they may load the blood with imperfectly prepared digestive products and putrefactive or poisonous results and also by their frequency and size, more or less rapidly deplete the blood current.

The doctor further says:

1. Green stools are never healthy stools.
2. They show imperfect digestion always.
3. In direct proportion with their presence is the damage and danger to the child.
4. These stools render children more susceptible to acute gastro-enteritis in hot weather.
5. The high infantile summer mortality follows children suffering from this colored stool.

6. Through unhealthy nutrition the blood is poisoned and the various tissues improperly nourished.

7. The excreting organs, particularly the kidneys and liver, are frequently damaged by the extraordinary duties imposed upon them in eliminating these poisonous results from the blood.

8. This continued irritation and innutrition favors the development of inherited diathesis and acquired cachexias.

9. No child is free from complications dangerous to life, or from developmental errors, who suffers from frequently recurring green-shaded stools, particularly the very liquid and foul-smelling ones.—*Charlotte Medical Journal.*

